# Pharmacokinetics, Brain Delivery, and Efficacy in Brain Tumor-Bearing Mice of Glutathione Pegylated Liposomal Doxorubicin (2B3-101)

**DOI:** 10.1371/journal.pone.0082331

**Published:** 2014-01-08

**Authors:** Pieter J. Gaillard, Chantal C. M. Appeldoorn, Rick Dorland, Joan van Kregten, Francesca Manca, Danielle J. Vugts, Bert Windhorst, Guus A. M. S. van Dongen, Helga E. de Vries, David Maussang, Olaf van Tellingen

**Affiliations:** 1 to-BBB technologies BV, Leiden, The Netherlands; 2 Department of Radiology and Nuclear Medicine, VU University Medical Center, Amsterdam, The Netherlands; 3 Blood-Brain Barrier Research Group, Molecular Cell Biology and Immunology, Neuroscience Campus Amsterdam, VU University Medical Center, Amsterdam, The Netherlands; 4 The Netherlands Cancer Institute - Antoni van Leeuwenhoek Hospital (NKI-AvL), Amsterdam, The Netherlands; Complutense University, Spain

## Abstract

Brain cancer is a devastating disease affecting many people worldwide. Effective treatment with chemotherapeutics is limited due to the presence of the blood-brain barrier (BBB) that tightly regulates the diffusion of endogenous molecules but also xenobiotics. Glutathione pegylated liposomal doxorubicin (2B3-101) is being developed as a new treatment option for patients with brain cancer. It is based on already marketed pegylated liposomal doxorubicin (Doxil®/Caelyx®), with an additional glutathione coating that safely enhances drug delivery across the BBB.

Uptake of 2B3-101 by human brain capillary endothelial cells *in vitro* was time-, concentration- and temperature-dependent, while pegylated liposomal doxorubicin mainly remained bound to the cells. *In vivo*, 2B3-101 and pegylated liposomal doxorubicin had a comparable plasma exposure in mice, yet brain retention 4 days after administration was higher for 2B3-101. 2B3-101 was overall well tolerated by athymic FVB mice with experimental human glioblastoma (luciferase transfected U87MG). In 2 independent experiments a strong inhibition of brain tumor growth was observed for 2B3-101 as measured by bioluminescence intensity. The effect of weekly administration of 5 mg/kg 2B3-101 was more pronounced compared to pegylated liposomal doxorubicin (p<0.05) and saline (p<0.01). Two out of 9 animals receiving 2B3-101 showed a complete tumor regression. Twice-weekly injections of 5 mg/kg 2B3-101 again had a significant effect in inhibiting brain tumor growth (p<0.001) compared to pegylated liposomal doxorubicin and saline, and a complete regression was observed in 1 animal treated with 2B3-101. In addition, twice-weekly dosing of 2B3-101 significantly increased the median survival time by 38.5% (p<0.001) and 16.1% (p<0.05) compared to saline and pegylated liposomal doxorubicin, respectively.

Overall, these data demonstrate that glutathione pegylated liposomal doxorubicin enhances the effective delivery of doxorubicin to brain tumors and could become a promising new therapeutic option for the treatment of brain malignancies.

## Introduction

Brain cancer is a devastating disease affecting many people worldwide. In the US, the incidence of primary brain tumors is 7–8 per 100,000, and it is expected that well over 23,000 new cases will be diagnosed in 2013 (http://www.cancer.gov/cancertopics/types/brain). In addition, metastatic brain tumors are more common than primary brain tumors. Independent whether the tumor has originated in the brain or metastasized from the periphery, the prognosis for patients is generally poor. The median survival after starting treatment, including surgery, radiation, chemotherapy, and combinations thereof, is less than a year [1], [2]. The standard treatment for primary brain tumors involves maximal surgical resection followed by chemoradiation therapy [3]. In contrast, the treatment of patients with metastases from peripheral tumors depends on the number of metastases: patients with multiple metastases are usually treated with whole-brain radiation therapy (WBRT), while patients with 3 or fewer metastases can benefit from aggressive local treatment with surgery or stereotactic radiosurgery (SRS) combined with WBRT [4].

Treatment of brain tumors with chemotherapy is limited due to the presence of the blood-brain barrier (BBB). This neuroprotective barrier regulates and maintains brain homeostasis, by actively excluding, effluxing and metabolizing potential neurotoxic compounds and by the selective directional transport of life-essential nutrients and metabolites between the systemic circulation and the brain. Although sometimes disputed, the current available evidence indicates that the BBB is still intact in small (metastatic) brain tumors, as well as in localized parts of larger tumors, thereby limiting the ability of chemotherapeutics to effectively reach brain tumors [5], [6], [7], [8]. Furthermore, even if detectable levels of drugs can be achieved in the brain, these drugs might not remain long enough and at a high enough concentration to ensure cancer cell apoptosis [9].

Brain drug delivery can be achieved in several ways. Direct administration into the brain using carmustine loaded wafers (Gliadel®) has been approved by the Food and Drug Administration (FDA) for the treatment of malignant glioma, providing about 3 weeks gain in overall survival [10], [11]. A major concern with this intervention is that it can only be done at one occasion and drug release from the wafer is a finite process. Moreover, direct administration only results in a localized delivery, which may not be suitable in case of a large and infiltrative tumor or when multiple tumors are located in the brain. Alternatively drugs can be delivered across the BBB; for example by disruption of the BBB by osmotic imbalance or vaso-active compounds, or by making use of endogenous transport systems [12], [13], [14]. Disruption of the BBB can however also cause neuronal damage due to unwanted macromolecules entering the brain and is considered highly invasive and painful, requiring specialized facilities and treatment conditions. Therefore, implementing a physiologically-based strategy appears to be a promising option for blood-to-brain drug delivery [13]. Glutathione, an endogenous tripeptide that possesses antioxidant-like properties, is actively transported across the BBB [15], [16], [17]. Although the molecular mechanism by which it is transported remains to be elucidated, we have shown that glutathione can be used as a targeting ligand coupled to pegylated liposomes to enhance drug delivery to the brain [18], [19].

Doxorubicin, an anthracycline antibiotic is among the most widely used anticancer agents. It inhibits the growth of many cancerous cell lines, including glioblastoma and breast cancer cell lines [20], [21]. It is currently on the market as free drug (Adriamycin®), encapsulated in conventional liposomes (Myocet®) and encapsulated in pegylated (“stealth”) liposomes (Doxil®/Caelyx®). The liposomal formulations have an improved safety profile with regard to cardiac or other toxicities, while having a similar efficacy [22]. As a free drug, doxorubicin has shown efficacy against malignant brain tumors when injected directly into the brain in the area where the tumor was located [23], [24], but not when injected intravenously [25]. Although von Holst *et al.* did find doxorubicin in brain tumor tissue, the amount was insufficient to exert an effect on brain tumor growth [25]. Doxorubicin encapsulated in pegylated liposomes showed a higher exposure after intravenous administration compared to free doxorubicin in a rat brain tumor model, as well as an increased life span [26], [27]. Moreover, in a small cohort of patients with recurrent glioma, pegylated liposomal doxorubicin was found safe and moderately effective after intravenous administration [28], [29], [30].

Based on the efficacy of pegylated liposomal doxorubicin against brain tumors and the ability of glutathione to safely enhance drug delivery to the brain, we have developed glutathione pegylated liposomal doxorubicin (2B3-101). In an *in vitro* model using human brain capillary endothelial cells the binding and uptake of 2B3-101 was determined and compared to non-targeted pegylated liposomal doxorubicin. Subsequently, using [^14^C]-labeled doxorubicin, we have investigated the pharmacokinetics (PK) and biodistribution of both glutathione-targeted and non-targeted pegylated liposomes in mice and compared this to free [^14^C]-doxorubicin. An additional PK and brain uptake study was performed with 2B3-101 and pegylated liposomal doxorubicin, to investigate the disposition of doxorubicin up to 4 days after administration. Finally, the efficacy of 2B3-101 was investigated in a mouse model of glioblastoma multiforma. Human glioblastoma cells (U87MG) were injected directly into the brain of athymic FVB mice, resulting in well-vascularized brain tumors that grow expansively to large lesions without necrosis [31]. The U87MG cells were transfected with luciferase for non-invasive follow-up of brain tumors by bioluminescence measurement of the brain tumors [31]. In the efficacy studies in the mouse model of glioblastoma multiforma, 2B3-101 was compared to free doxorubicin and pegylated liposomal doxorubicin. Initially a dosing regimen of 5 mg/kg doxorubicin equivalents once weekly was selected, which was based on previous experience considered to be the maximum tolerable dosing regimen for free doxorubicin. Since this dosing regime was well tolerated for the pegylated liposomal doxorubicin and 2B3-101, the dosing frequency of these formulations was increased to twice weekly in the second experiment. This second experiment was performed to confirm the positive results obtained in the first run and to generate the survival curves for the different treatments.

## Methods

### Liposomes

**Figure 1:**
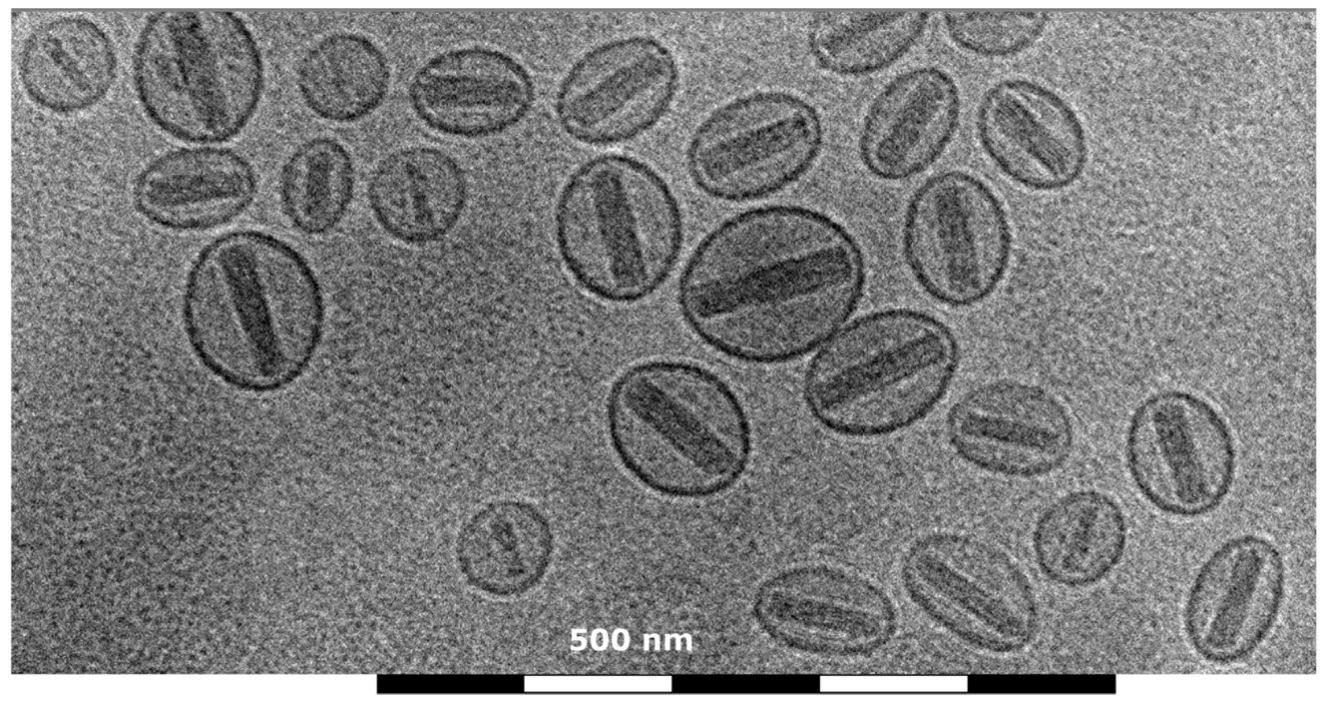
Representative cryo-electron microscopy image of 2B3-101. This image (30,000× magnified) shows the typical coffee bean structure of precipitated doxorubicin inside the liposomes.

Glutathione pegylated liposomal doxorubicin (2B3-101) was prepared in our laboratories according to the commercial Doxil/Caelyx preparation method, i.e. using active doxorubicin loading against an ammonium sulfate gradient [32]. To have the optimal comparator, pegylated liposomal doxorubicin was also prepared in our laboratories using the exact same methods and constituents, except for the addition of glutathione. In short, glutathione (Sigma-Aldrich, Zwijndrecht, the Netherlands) and N-[(3-Maleimide-1-oxopropyl)aminopropyl polyethyleneglycol_2000_-carbamyl] distearoylphosphatidyl-ethanolamine (DSPE-PEG-maleimide, NOF, Grobbendonk, Belgium) were incubated at a 1∶1 molar ratio for 2 hours (h) at room temperature, before adding the resulting glutathione-PEG micelles to a pre-heated ammonium sulfate solution (250 mM in MilliQ; Sigma-Aldrich, Zwijndrecht, the Netherlands). For non-targeted pegylated liposomes, micelles were made by addition of methoxyPEG_2000_-DSPE (mPEG-DSPE, Lipoid, Cham, Switzerland) to the ammonium sulfate solution. Hydrogenated soy phosphatidylcholine (HSPC, Lipoid, Cham, Switzerland) and cholesterol (Sigma-Aldrich, Zwijndrecht, the Netherlands) (60 and 40% of total lipids, respectively) were dissolved in ethanol, added slowly to the ammonium sulfate solution, and incubated for 30 minutes (min) at 60°C. Five percent GSH-PEG-DSPE (for 2B3-101) or mPEG-DSPE (for non-targeted pegylated liposomes) of total lipids was added to prepare the liposomes. After extrusion through 200/200 and 100/80 nm filters, the liposomes were dialyzed against PBS containing 9.4% sucrose. Doxorubicin (2 mg/ml; gift from TTY Biopharm, Taipei, Taiwan) in 9.4% sucrose was added to the liposomes and incubated for 30 min at 60°C. Finally, histidine (1.55 mg/ml; Sigma-Aldrich, Zwijndrecht, the Netherlands) was added and after a short incubation at 60°C and at 0°C, 2B3-101 was stored at 2–8°C. Mean liposome size was 95 nm (polydispersity index <0.1; indicative of a narrow size distribution) and contained 2 mg/ml doxorubicin; more than 90% of which was encapsulated in the liposomes (Figure 1). For the PK and biodistribution study at VUmc, [^14^C]-labeled doxorubicin was used at a tracer amount and mixed with doxorubicin before active loading. All other steps in the preparation protocol were the same.

### Cell culture

The human brain endothelial cell line hCMEC/D3 was a gift from Pierre-Olivier Couraud (Institut Cochin, Paris, France). The hCMEC/D3 cell line has been generated from a primary culture of human adult brain endothelial cells immortalized via lentiviral transduction of human telomerase reverse transcriptase (hTERT) and simian virus 40 (SV40) large T antigen [33]. Cells were grown on collagen type I-coated plates in endothelial cell basal medium-2 supplemented with human epithelial growth factor (EGF), hydrocortisone, gentamicin, amphotericin-B, vascular endothelial growth factor (VEGF), human fibroblast growth factor (FGF)-B, R3 insulin-like growth factor (R3-IGF)-1, ascorbic acid, and 2.5% fetal bovine serum (Lonza, Verviers, Belgium).

### *In vitro* uptake experiments

hCMEC/D3 cells were seeded in 24-wells plates to reach confluence 3 days later (start of the experiment). To determine concentration-dependent binding (4°C) and uptake (37°C), cells were incubated with 0–450 µg/mL 2B3-101 or pegylated liposomal doxorubicin (concentration based on HSPC lipid concentration) for 5.5 h. For time-dependent uptake studies, cells were incubated with 450 µg HSPC per mL of both liposomal formulations for 1–5.5 h. After incubation, cells were washed with cold PBS, harvested, fixed, and analyzed by flow cytometry using a FACScalibur (BD Biosciences, Breda, the Netherlands). Data were analyzed by selecting the cell population based on the forward (FSC) and side scatter (SSC) plot and subsequently quantifying their mean fluorescence intensity (MFI) in the FL2 channel, indicative of the amount of doxorubicin present on/in the cells. To calculate the percentage of internalized liposomes the MFI at 37°C (binding and uptake) was corrected for the MFI at 4°C (binding) and divided by the MFI at 4°C: (MFI@37°C−MFI@4°C)/MFI@4°C. A paired t-test on the mean results from 3 independent experiments, each performed in triplo, was subsequently performed.

### Animals

#### Ethics statements

For the pharmacokinetic and biodistribution study at the VUmc, Amsterdam, the Netherlands, 8–10 week old female athymic FVB mice were obtained from Harlan CPB (the Netherlands) and maintained in filtertop cages with free access to food and water. All animal experimental procedures were conducted according to the standard operating procedures of the lab animal facility and were approved by the Animal Ethics Committee of VUmc (Permit number KNO 10-03).

For the pharmacokinetic and biodistribution study at the Netherlands Cancer Institute (NKI, Amsterdam, the Netherlands), female athymic FVB mice were bred at the NKI and maintained in individually ventilated cages (IVC) with free access to food and water. All animal experimental procedures were conducted according to the standard operating procedures of the lab animal facility and were approved by the Animal Ethics Committee of the NKI (Permit number NKI 08.022).

For the efficacy studies, female athymic FVB mice were bred at the NKI and maintained in IVC with free access to food and water. All animal experimental procedures were conducted according to the standard operating procedures of the lab animal facility and were approved by the Animal Ethics Committee of the NKI (Permit number NKI 08.022).

### Pharmacokinetics and biodistribution

Animals (6 per group) received 1 intravenous (IV) injection through the tail vein of 2B3-101 with [^14^C]-labeled doxorubicin, non-targeted pegylated liposomal [^14^C]-labeled doxorubicin, or free [^14^C]-labeled doxorubicin (all at a doxorubicin dose of 5 mg/kg). To determine the pharmacokinetics (PK) blood samples were taken at 15, 30, 60, 90, 180, 300 and 1260 min after injection. Animals were sacrificed 1260 min after injection, perfused with PBS, and [^14^C]-doxorubicin was determined in the following tissues: brain, skin, heart, lung, liver, spleen, kidney, bladder and muscle. Tissue samples were dissolved in Soluene-350 (Packard Instrument Company, Groningen, the Netherlands), and heated at 50°C for 24 h. After decolorization with 30% H_2_O_2_, Ultima Gold Liquid scintillation mixture was added and radioactivity was determined in an LKB-Wallac 1410 Liquid Scintillation Counter (Pharmacia, Woerden, the Netherlands). Results were presented as % of injected radioactivity dose per mL blood or per gram tissue (%ID/mL or %ID/gram) to account for the differences between injected [^14^C] radioactivity. The area under the curve (AUC) was calculated by the linear trapezoidal rule up to the last sampling time point. The differences between the liposomal treatments in the concentration-time curves were analyzed using an unpaired two-tailed t-test, both for the AUC as well as for each of the time points a plasma sample was taken. The differences in biodistribution between all 3 treatments were analyzed using a one-way ANOVA with a Tukey post-test. The analysis of plasma samples showed that the administration of test compounds did not succeed in 2 mice (one in the 2B3-101 and one in the non-targeted pegylated liposomal doxorubicin group), these were excluded in the results and further analysis.

In addition, a PK and brain uptake study in 8–10 week old mice was performed at the NKI focusing on the disposition of 2B3-201 and pegylated liposomal doxorubicin up to 4 days after injection. Mice (n = 3 per group per time point) were IV injected with 2B3-101 or pegylated liposomal doxorubicin (5 mg/kg). Blood samples were drawn at 4, 24, and 96 h and the brains of the non-perfused animals were isolated. Doxorubicin concentrations in plasma and tissue homogenates were determined using HPLC with fluorescence detection [34]. Statistical analysis was performed using an unpaired two-tailed t-test, focusing on the results obtained 96 hours after administration.

### Efficacy in a mouse model of glioblastoma multiforme

Brain tumors were initiated using the cerebral injection procedure as described previously [31]. In short, mice (7–10 weeks old) were anaesthetized and placed in a stereotactic frame. A hole of 1 mm in diameter was drilled at 2 mm lateral and 1 mm anterior to the bregma. Human U87MG cells stably transfected with luciferase (U87MG-luc) were resuspended as single cells in Hanks Balanced Saline Solution (HBSS) and injected using a 30-gauge needle inserted 3 mm below the skull surface; 2 µl containing 1·10^5^ cells was injected at a rate of 0.5 µl/min.

Tumor growth was visualized and quantified twice weekly using an IVIS imaging system similar to the method described by Kemper *et al.* [31]. Animals were anaesthetized and luciferin (150 mg/kg) was injected intraperitoneally (IP) 15 min prior to imaging. The mice were photographed while placed on their front and the bioluminescence intensity (BLI) was measured in the region of interest (ROI). The BLI value just prior to the initiation of the treatment was used to stratify animals in each of the treatment groups and as reference value to calculate the %BLI for each individual animal [35]. These normalized effects were subsequently LOG-transformed to render a Gaussian distribution of the effect measurement (expressed as log%BLI) to allow for subsequent two-way ANOVA testing, determining the effects between different treatment groups in time. The mean log%BLI data were then back converted into mean %BLI and plotted for graphical outcome display.

Treatments were started at 12–14 days after tumor cell injections, when tumors were well established (i.e. tumors had started the exponential growth phase as examined by bioluminescence). All drug administrations were given by IV injection into the lateral tail vein. In the first experiment, animals (n = 9 per treatment group) received saline or 5 mg/kg doxorubicin equivalents once weekly in the form of free doxorubicin, pegylated liposomal doxorubicin, or 2B3-101. Injections were given on days 12, 19 and 26 after tumor initiation, and animals were sacrificed on a preset study end date (day 29 after tumor initiation). In the second experiment survival was included as an endpoint and the dose was doubled; animals received saline (n = 14) or 5 mg/kg doxorubicin equivalents twice weekly as pegylated liposomal doxorubicin (n = 10) or 2B3-101 (n = 10) on days 14, 17, 21 and 24 after tumor initiation. Treatment with two weekly doses of 5 mg/kg free doxorubicin was not included, as the animals would not tolerate this increased dose.

In both experiments animals were monitored and weighed daily. Animals were sacrificed when they lost more than 20% of their body weight or if they showed other signs of unacceptable toxicities. The number of animals throughout both experiments are summarized in Table S1.

### Statistical analyses

All data are presented as means with standard error of the mean (SEM). Statistical analyses were performed using Prism 4.0 (GraphPad Software, La Jolla, CA). In each experiment it is indicated which statistical analysis is performed. When comparing 2 treatment groups, a two-tailed t-test was used. When comparing 3 or more groups, a one-way ANOVA was used together with a Tukey post-test to compare all treatment groups to each other. For the efficacy studies in the mouse model of glioblastoma multiforme, a two-way ANOVA analysis was used to determine the effects between different treatment groups in time.

## Results

### Human brain capillary endothelial cells specifically take up 2B3-101

**Figure 2:**
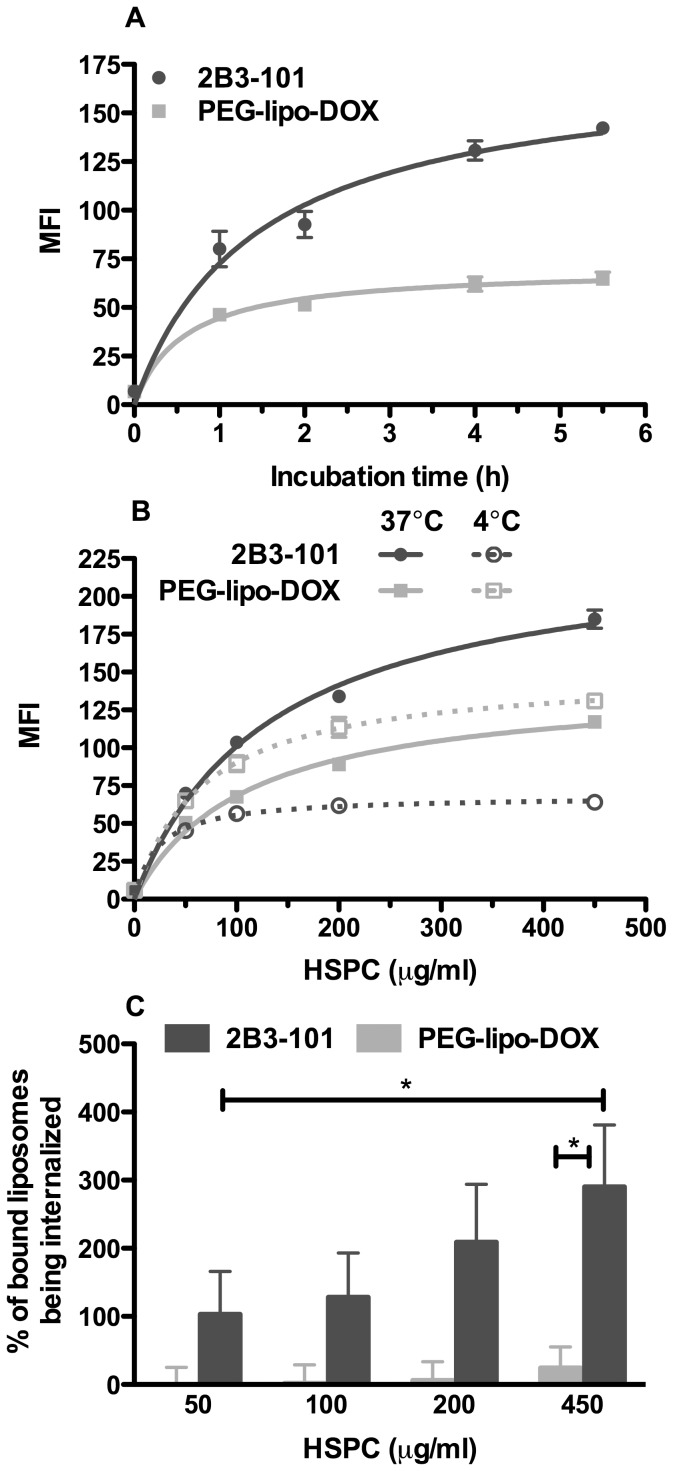
Uptake of 2B3-101 in human brain endothelial hCMEC/D3 cells. (A) Representative graph (mean ± SEM, n = 3 per time point) of the time-dependent experiment at 37°C showed a higher uptake of 2B3-101 compared to pegylated liposomal doxorubicin (PEG-lipo-DOX), both at a concentration of 450 µg/ml HSPC. (B) Representative graph of the concentration-dependent experiments at 4 and 37°C (mean ± SEM, n = 3 per concentration). Cells incubated at 4°C (dotted lines) show a higher fluorescence when treated with pegylated liposomal doxorubicin than with 2B3-101. However, at 37°C (full lines), 2B3-101 incubation resulted in a stronger fluorescence than pegylated liposomal doxorubicin. (C) The percentage of bound 2B3-101 being internalized [(MFI@37°C−MFI@4°C)/MFI@4°C] is increasing in a concentration-dependent manner for 2B3-101, while pegylated liposomal doxorubicin mainly remains bound to the cells. The overall mean ± SEM from 3 independent experiments (each n = 3) was used in a paired t-test. *p<0.05 2B3-101 vs. PEG-lipo-DOX, both at 450 µg/ml HSPC and 2B3-101 at 450 µg/ml vs. 50 µg/ml.

To investigate the ability of 2B3-101 and conventional pegylated liposomal doxorubicin to target the BBB, we have used an *in vitro* model of human brain capillary endothelial cells. After incubation of hCMEC/D3 cells with medium or liposomal doxorubicin formulations and FACS analysis, cells were gated based on the FSC/SSC plots (Figure S1). Using a maximum concentration of liposomes (450 µg/ml) and incubation time (5.5 h), FSC/SSC scatters were similar between liposomal doxorubicin- and medium-treated cells, suggesting a lack of toxicity of both 2B3-101 and pegylated liposomal doxorubicin with these concentration and incubation time. Subsequently, the fluorescence of cells exposed to 2B3-101 was followed in a time-dependent manner and was higher compared to that of cells incubated with pegylated liposomal doxorubicin. While the signal from 2B3-101-treated cells did not reach a plateau within 5.5 h, the level of pegylated liposomal doxorubicin signal already plateaued after 2–4 h (Figure 2A). We then measured the non-selective binding of liposomes onto hCMEC/D3 cells at 4°C: non-targeted pegylated liposomal doxorubicin incubation resulted in a 2-fold higher fluorescence signal compared to 2B3-101, indicative of more extensive interaction of pegylated liposomal doxorubicin on the outer surface of the cells (Figure 2B). Incubation of the cells at 37°C with concentrations ranging between 50 and 450 µg/mL HSPC consistently showed a higher fluorescent signal for 2B3-101 compared to pegylated liposomal doxorubicin (Figure 2B). Likewise, the percentage of internalized liposomes was higher for 2B3-101 compared to pegylated liposomal doxorubicin at all the concentrations that were tested, demonstrating an improved targeting into brain endothelial cells for 2B3-101 (Figure 2C).

### 2B3-101 has a prolonged circulation time and enhanced brain delivery

**Figure 3:**
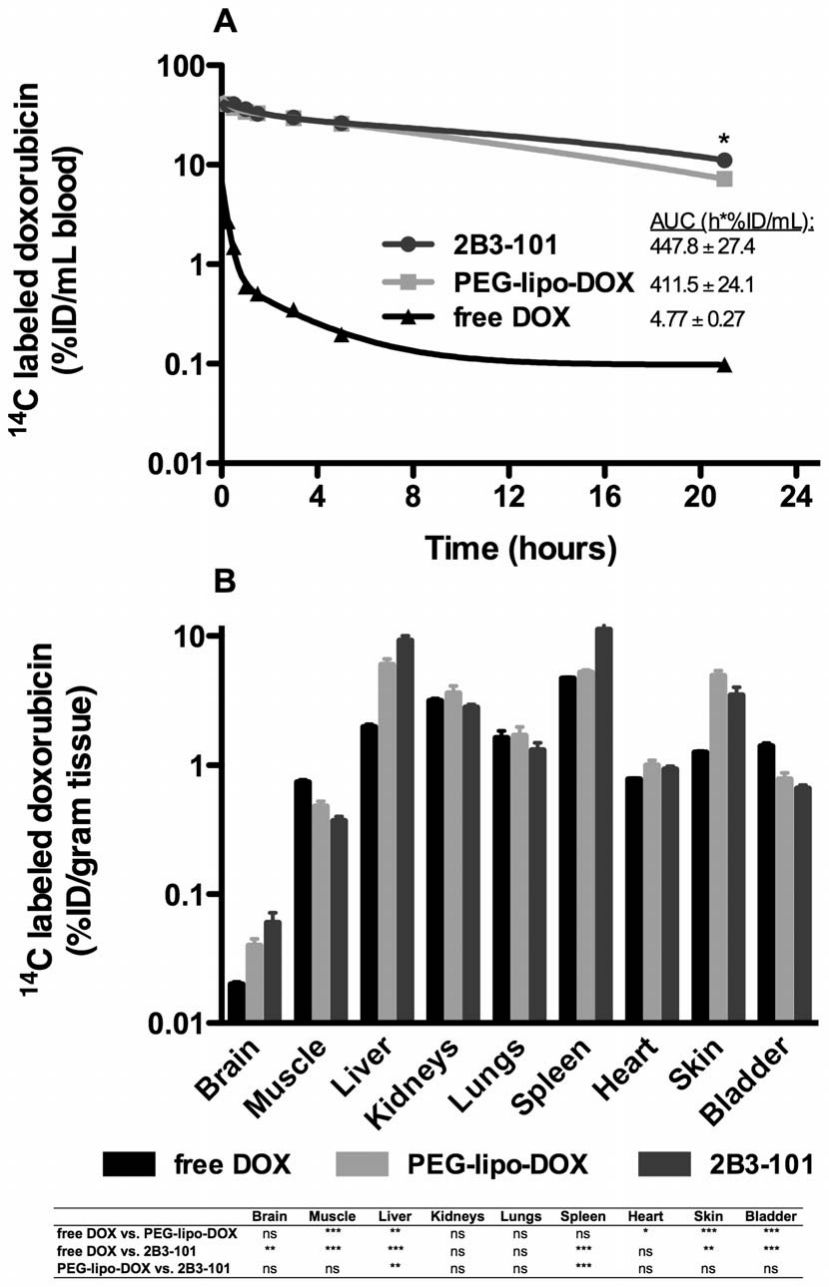
Pharmacokinetics and biodistribution of 2B3-101, pegylated liposomal doxorubicin and free doxorubicin in mice. Healthy mice (n = 5–6 per group) received 1 dose (5 mg/kg) 2B3-101, pegylated liposomal doxorubicin (PEG-lipo-DOX) or free doxorubicin (free DOX). (A) The pharmacokinetic profile shows a higher exposure for 2B3-101 and pegylated liposomal doxorubicin compared to free doxorubicin. While no statistical difference was observed between the AUC of PEG-lipo-DOX and 2B3-101, a significant difference in plasma concentrations was observed 21 hours after administration (p = 0.0187, unpaired two-tailed t-test). (B) The biodistribution study shows that 2B3-101 administration results in the highest brain concentration of doxorubicin. A summary of the one-way ANOVA using a Tukey post-test is given below the graph. %ID: % of injected dose.

By using [^14^C]-labeled doxorubicin, doxorubicin levels in plasma and tissues could be realistically estimated without the need of purification of the samples. Both liposomal formulations had a similar PK profile (Figure 3). The area under the curve (AUC) for 2B3-101 and pegylated liposomal doxorubicin was 447.8±27.4 and 411.5±24.1 h*%ID/mL, respectively (p = 0.35, unpaired two-tailed t-test). Administration of free doxorubicin resulted in a lower plasma exposure (AUC: 4.77±0.27 h*%ID/mL; no statistical analysis performed). Tissue analysis in these perfused animals showed that the highest concentrations for all 3 dosing forms of doxorubicin were reached in liver, spleen, and kidney, followed by skin, lung, heart, bladder and muscle. In kidneys and lungs no significant differences between the 3 treatments were observed (one-way ANOVA with Tukey post-test). Analysis of brain tissue showed that free doxorubicin did reach the brain (0.02 %ID/gram tissue). Highest doxorubicin concentrations were found in animals that received 2B3-101 (p = 0.0039, one-way ANOVA including Tukey post-test), approximately 3-fold more compared to free doxorubicin (P<0.01, according to the Tukey post-test) and 1.5-fold more compared to pegylated liposomal doxorubicin (not significant according to the Tukey post-test).

**Figure 4:**
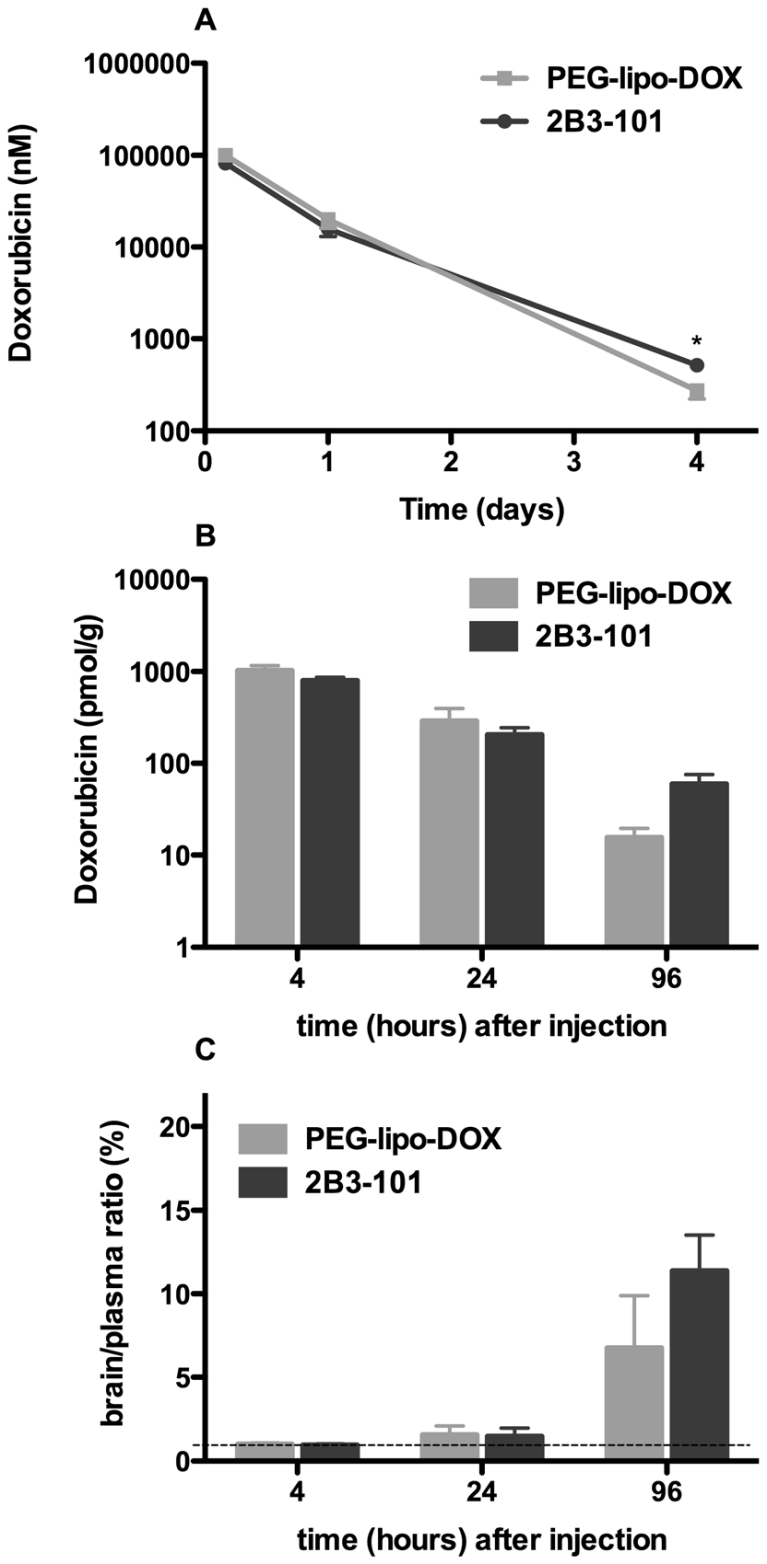
Plasma pharmacokinetics and brain uptake of 2B3-101 and pegylated liposomal doxorubicin in healthy mice. (A) Plasma concentration after 1 IV dose (5 mg/kg in 3 mice per group per time point) decreases with time for both liposomal formulations. After 4 days, less than 1% of the amount measured in plasma at 4 h after administration was present in the circulation. (B) The brain concentration decreases in time in line with the plasma level. (C) The brain∶plasma ratio shows retention in brain 4 days after administration of both liposomal formulations. The dotted line at a ratio of 1.4, indicates that the amount in brain equals the amount originating from blood [36]. Mean concentrations ± SEM are plotted in all graphs. Statistical analysis using an unpaired two-tailed t-test showed that there was a significant difference in the plasma concentrations of 2B3-101 and PEG-lipo-DOX (p = 0.021) 4 days after administration, but not in the brain concentrations (p = 0.052), and not in the brain∶plasma ratios (p = 0.29).

Next, a 4-day pharmacokinetics study of doxorubicin using HPLC quantification was performed following a single dose of 2B3-101 or pegylated liposomal doxorubicin and showed a comparable plasma profile for both formulations (Figure 4A). The area under the curve (AUC) was 73.2±5.5 and 90.0±8.6 days*nmol/mL for 2B3-101 and pegylated liposomal doxorubicin, respectively (not significant, unpaired two-tailed t-test). Brain exposure of both formulations was demonstrated at all time points (Figure 4B). At the early time-points (4 and 24 h), the doxorubicin concentration in brain homogenates was similar after administration of 2B3-101 and pegylated liposomal doxorubicin, which was in line with the plasma concentrations. Four days post-injection, the brain doxorubicin concentration trended to be higher for 2B3-101 (p = 0.052, unpaired two-tailed t-test; Figure 4B), which was in line with the plasma concentrations (p = 0.021). Likewise, when calculating the brain to plasma ratio for doxorubicin, a trend towards a higher retention of 2B3-101 was observed 4 days after injection (Figure 4C; p = 0.29), which was not shown at the earlier time-points. At 4 and 24 h after drug administration the brain level approximated the fraction originating from blood (brain∶plasma ratio of 1.4% [36]; Figure 4C).

### 2B3-101 has an acceptable safety profile

**Figure 5:**
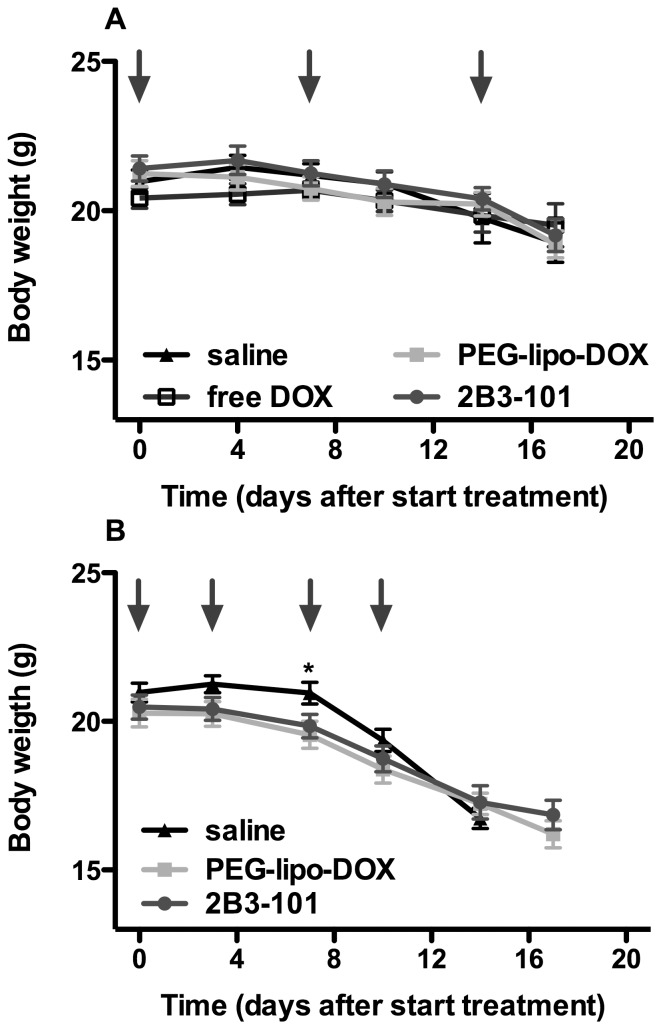
Body weight of animals with experimental brain tumors. (A) Animals (n = 9 per group) received once-weekly IV administrations of saline, 2B3-101, pegylated liposomal doxorubicin (PEG-lipo-DOX) or free doxorubicin (free DOX), all at a 5 mg/kg doxorubicin equivalent. (B) Treatment was increased to 5 mg/kg doxorubicin equivalents twice weekly (saline: n = 14; PEG-lipo-DOX and 2B3-101 treatment groups: n = 10). The mean ± SEM is plotted; the arrows indicate IV injections. Statistical analysis using a one-way ANOVA with Tukey post-test was performed at each time point. * saline vs. PEG-lipo-DOX p<0.05, saline vs. 2B3-101 no significant difference, 2B3-101 vs. PEG-lipo-DOX no significant difference.

Throughout the efficacy studies in tumor-bearing mice, animals were carefully monitored to also evaluate the tolerability of the treatment. 2B3-101 or pegylated liposomal doxorubicin, either at a weekly or at a twice-weekly dose of 5 mg/kg did not show major injection-related adverse events. Moderate to severe skin reactions (rash) were noted under the animal welfare monitoring program and confirmed by the investigators in all animals treated with pegylated liposomal doxorubicin or 2B3-101, but not in animals treated with free doxorubicin. Furthermore, no neurological symptoms were observed during both studies. The increase in dosing frequency did result in a stronger decrease in bodyweight in all treatment groups; most tumor-bearing animals treated once weekly had lost 8% of their body weight three days after the last dosing, which was increased to approximately 18% when treatment was given twice weekly (Figure 5). Also, after the first week of treatment with 2×5 mg/kg/week dosing, a lower body weight was observed when compared to saline (p = 0.04, one-way ANOVA with Tukey post-test at day 7) for pegylated liposomal doxorubicin (p<0.05 vs. saline, according to the Tukey post-test) and 2B3-101 (no significant difference vs. saline, according to the Tukey post-test). This is most likely due to the increased dose-intensity in the biweekly doxorubicin schedule.

### 2B3-101 reduces brain tumor growth and prolongs survival

**Figure 6:**
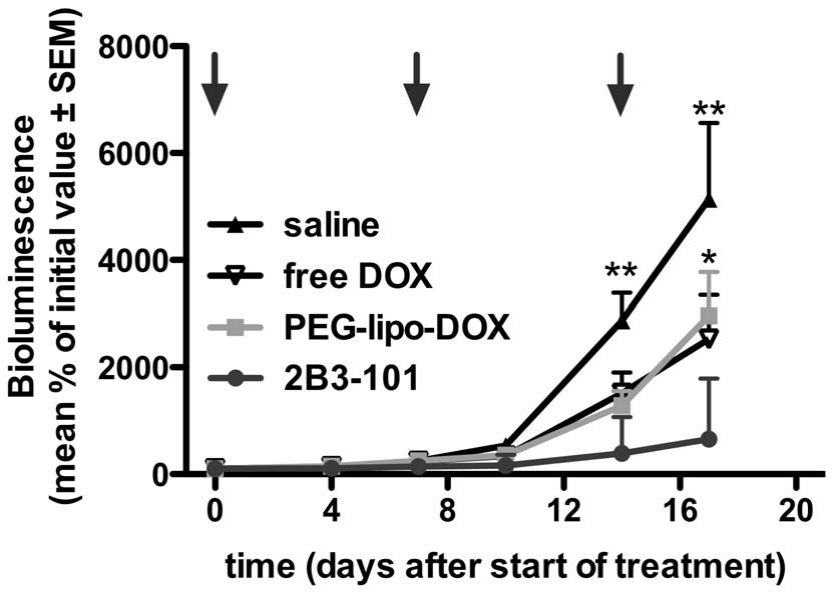
Inhibition of brain tumor growth by 2B3-101 in mice with experimental glioblastoma. Animals (n = 9 per group) received once-weekly IV administrations of saline, 2B3-101, pegylated liposomal doxorubicin (PEG-lipo-DOX) or free doxorubicin (free DOX), all at a 5 mg/kg doxorubicin equivalent. The study was ended at the pre-set study endpoint. Brain tumor growth was measured by bioluminescence (BLI). Arrows indicate IV injections. *P<0.05 2B3-101 vs pegylated liposomal doxorubicin (PEG-lipo-DOX); **P<0.01 2B3-101 vs saline (two-way ANOVA on Log%BLI).

**Figure 7:**
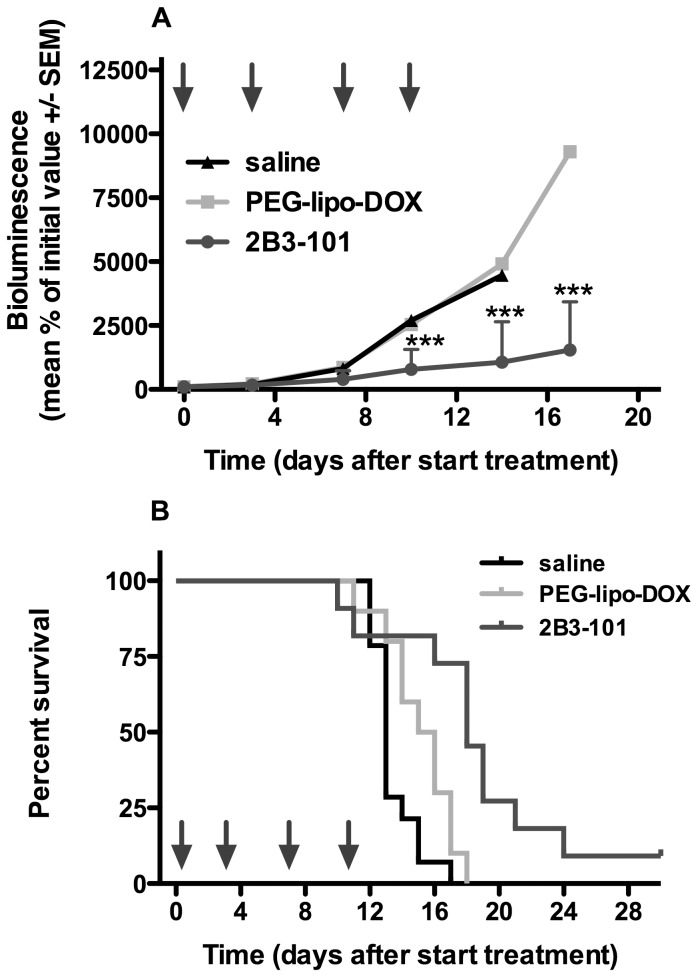
Efficacy of 2B3-101: Inhibition of brain tumor growth and increased survival of mice with experimental brain tumors. Animals received twice-weekly IV administrations of saline (n = 14), 2B3-101 (n = 10), or pegylated liposomal doxorubicin (PEG-lipo-DOX; n = 10), all at 5 mg/kg doxorubicin equivalents. (A) Brain tumor growth was measured by bioluminescence (BLI). Arrows indicate IV injections. ***P<0.001 2B3-101 vs saline and pegylated liposomal doxorubicin (two-way ANOVA on Log%BLI). (B) Survival of animals was increased from 13 days (median survival time after treatment with saline) to 15.5 days after treatment with PEG-lipo-DOX (p<0.05 compared to saline) and 18 days after treatment with 2B3-101 (p<0.05 and p<0.001 compared to pegylated liposomal doxorubicin and saline, respectively).

The efficacy of 2B3-101 was investigated in mice with experimental brain tumors. Weekly treatment with 5 mg/kg doxorubicin or pegylated liposomal doxorubicin resulted in an inhibition of brain tumor growth compared to saline, albeit not significant (Figure 6). In contrast, treatment with 2B3-101 at the same dose did result in a significant inhibition of tumor growth (P<0.05 vs pegylated liposomal doxorubicin and P<0.01 2B3-101 vs saline). Moreover, 2 from the 9 animals receiving 2B3-101 showed a complete regression (accounting for the large standard errors in Figure 5), which was not observed in the other treatment groups. Doubling the dosing frequency to 5 mg/kg twice weekly resulted in a even more significant reduction in brain tumor growth after treatment with 2B3-101 compared to saline and pegylated liposomal doxorubicin (P<0.001; Figure 7A). Again, a complete regression was observed in 1 animal treated with 2B3-101.

For ethical reasons, the first experiment with weekly treatments was not designed as a survival experiment; animals were terminated at the preset study end date. However, while none of the animals in the pegylated liposomal doxorubicin and 2B3-101 treatment groups were terminated before the end of the study, 3 mice in the saline group and 2 in the doxorubicin treatment group were terminated during the course of the experiment after their body weight had dropped by more than 20%. This decrease in body weight was not considered to be due to the treatment, but to brain tumor growth. Since the liposomal formulations of doxorubicin were well tolerated and effective, a subsequent confirmatory survival study was initiated at a more intense dosing regimen. Treatment with 5 mg/kg 2B3-101 twice weekly resulted in a significant survival benefit when compared to the control groups (Figure 7B). An increase in median survival time with 2B3-101 of 38.5% as compared to saline (p<0.001) and 16.1% compared to pegylated liposomal doxorubicin (p<0.05) was observed. The small survival time benefit of pegylated liposomal doxorubicin treatment over saline was also statistically significant (p<0.05).

## Discussion

The neuroprotective BBB limits the treatment options for brain tumors. By combining an existing drug that is able to inhibit tumor growth, i.e. pegylated liposomal doxorubicin, with a glutathione-based brain drug delivery strategy, we were able to show that 2B3-101 showed consistently better outcomes compared to conventional pegylated liposomal doxorubicin.

*In vitro*, 2B3-101 was actively taken up by the brain capillary endothelial cells in a concentration- and time-dependent manner (Figure 2). The concentration used for the time-dependent experiments was 450 µg/ml based on HSPC, which relates to 92 µg/ml doxorubicin. In the *in vivo* PK study, approximately 50 µg/ml liposomal doxorubicin was measured 4 h after administration. The concentrations used in the *in vitro* studies were, therefore, comparable to the initial time points of the *in vivo* studies. Compared to 2B3-101, non-targeted pegylated liposomal doxorubicin showed a higher binding capacity to brain capillary endothelial cells *in vitro* at 4°C, while at 37°C (binding+uptake) a profound increase in signal was observed only for 2B3-101. The charge of the liposomal formulations may be a reason for the higher binding of pegylated liposomal doxorubicin as it is slightly less negatively charged (zeta potential of −4.3 mV), compared to 2B3-101 (zeta potential of −8.3 mV), therefore exerting less electrostatic repulsion with the negatively-charged cellular glycocalyx. The specific binding and active uptake of 2B3-101 in the brain capillary endothelial cells demonstrates the GSH-mediated targeting properties to the BBB *in vitro*.

The *in vitro* results were confirmed *in vivo*, where brain homogenate levels of doxorubicin were highest after administration of 2B3-101 compared to pegylated liposomal doxorubicin and free doxorubicin. For the short-term experiment with [^14^C]-labeled doxorubicin, the animals were perfused with PBS before harvesting tissues for the analysis of brain uptake. Since perfusion will never completely remove all blood from blood vessels inside the tissue specimens and can result in variability between samples [37], there will still remain plasma-associated doxorubicin in brain homogenates. Especially for long-circulating pegylated liposomes with a half-life of several days, the plasma concentration at 21 h after administration is still relatively high. Therefore, quantification of brain uptake of doxorubicin could be confounded due to a variable degree of plasma concentration [37]. In the 4-day PK and brain uptake study animals were not perfused, therefore, plasma-associated doxorubicin will have a strong influence on the interpretation of the brain exposure data. Dai et al. [36] have determined that 1.4% of the non-permeable BBB marker inulin was present in homogenates of non-perfused brain samples. At 4 and 24 h after administration our results approximate this brain∶plasma ratio of 1.4%, while 4 days after administration a higher brain retention of 2B3-101 compared to pegylated liposomal doxorubicin was observed. To further investigate the brain uptake of 2B3-101 and compare this to non-targeted pegylated liposomal doxorubicin in a separate study submitted for publication elsewhere, a cerebral open-flow microperfusion study (cOFM) was carried out, since the more classical microdialysis method is not suitable for lipophilic compounds such as doxorubicin [38]. The cOFM study in rats showed an almost 5-fold higher brain uptake of doxorubicin after administration of 2B3-101 compared to pegylated liposomal doxorubicin (*unpublished data*).

In addition to increasing uptake by brain endothelial cells *in vitro*, the addition of glutathione to pegylated liposomal doxorubicin improved the therapeutic effect as 2B3-101 significantly enhanced inhibition of brain tumor growth *in vivo* compared to pegylated liposomes (Figure 6). This beneficial effect was confirmed when the dosing frequency was increased from a once-weekly dose of 5 mg/kg to a twice-weekly dose (Figure 7A). Furthermore, median survival time of mice receiving twice-weekly injections of 2B3-101 improved by 38.5% compared to control (Figure 7B). Three animals treated with 2B3-101 even showed a complete regression (2 in the once-weekly treatment group, 1 in the twice-weekly treatment group). The difference in efficacy between pegylated liposomal doxorubicin and 2B3-101 are not due to differences in systemic exposure as the pharmacokinetic profiles and AUCs were not significantly different (Figures 3, 4). In addition, the systemic accumulation of doxorubicin in the twice-weekly dosing scheme was negligible, as more than 99% of doxorubicin was cleared before the next injection (4 days later).

The brain tumor model used in this study (U87MG cells) has a relatively leaky BBB [31]. Still the efficacy of non-targeted pegylated liposomal doxorubicin on brain tumor growth and survival was small, whereas the efficacy of 2B3-101 at a similar dose was significantly more pronounced. This indicates that glutathione as targeting ligand does have added therapeutic value. Recent studies with pegylated liposomal doxorubicin in other brain tumor models showed that non-targeted pegylated lipsomal doxorubicin at comparable doses to our study did prolong survival of animals by several days [39], [40]. However, combining pegylated liposomal doxorubicin with ultrasound-induced disruption of both the BBB and the blood-tumor barrier resulted in a 100% increase of median survival time compared to non-treated animals [40]. This indicates once more that the barriers in the brain as well as in the tumor are impermeable for treatment. An active targeting molecule, such as glutathione, is necessary to improve treatment outcomes. In addition to the current work in brain tumors, other studies with glutathione pegylated liposomes using different encapsulated drug molecules have indicated an improved efficacy (in an model of neuroinflammation [18]) or enhanced brain delivery (in 2 microdialysis studies [19], [41]), all with intact BBB.

Endogenous glutathione is present in the human body and brain as one of the antioxidants, protecting against reactive oxygen species. For this reason it is also used as supportive therapy in chemotherapy [42]. However, part of the mechanism of action of doxorubicin may be the production of reactive oxygen species (ROS) [43], which could be reduced by the addition of glutathione. Our data, however, provide no indication that glutathione conjugated to PEG will decrease the efficacy of doxorubicin. On the one hand, the effect on ROS is most likely limited because the free thiol group of glutathione is used for the conjugation. On the other hand, even if GSH-PEG would be recycled in the endogenous pool of glutathione, the low (micromolar) concentrations used for drug targeting will not affect the physiological plasma and brain concentrations, which are in the millimolar range [13].

Treatments with pegylated liposomal doxorubicin and 2B3-101 were well tolerated. In both efficacy studies, animals were carefully and frequently monitored for side effects. This study was not designed as toxicity study; therefore, no clear dose-dependent toxicity could be determined. All deaths were due to tumor progression in the brain. The U87MG intracranial glioma model is a very reproducible model. Animals behave relatively normal until a few days before death when they start losing body weight due to the progressive deterioration of their condition [31]. Moderate to severe skin reactions were observed with 2B3-101 and pegylated liposomal doxorubicin, but not with free doxorubicin, which was in line with findings in a clinical setting [44]. The addition of glutathione did not change the safety of pegylated liposomal doxorubicin as moderate to severe skin reactions were also observed in this treatment group. No injection-related adverse events were observed in any of the treatment groups and animals did not show any neurological symptoms. Animals did loose weight after treatment with pegylated liposomal doxorubicin or 2B3-101 (Figure 5), but this was comparable to the weight loss in the control group and was thus not considered treatment-related.

Although numerous drugs are being investigated for the treatment of brain cancer, the success rate is to date limited. With regard to brain-targeted therapy, ANG1005 is being evaluated in clinical trials. This molecule consists of a brain-targeting peptide (Angiopep-2, 19 amino acids) conjugated with 3 molecules of paclitaxel. In a preclinical study, mice inoculated with U87MG cells were treated with ANG1005 (50 mg/kg); treatment started 3 days after inoculation of the tumor cells and was repeated every 3 days up to day 15 (5 IV injections in total) [45]. The median survival time increased from 16.5 days (saline) to 19 days (ANG1005; p = 0.02). The results for free (unconjugated) paclitaxel were not reported in this tumor model with a leaky BBB. However, in our hands paclitaxel given at a lower dose of 20 mg/kg/week ×2 shows already a modest efficacy against U87 (*unpublished data*). Angiopep-2 is also conjugated to doxorubicin and etoposide. Both showed an increased brain and brain tumor uptake when compared to the native compounds [46]. Thus far, however, no efficacy studies with doxorubicin and etoposide conjugates have been published. ANG1005 has been clinically investigated in patients with advanced solid tumors; it was well tolerated and preliminary efficacy was shown in patients with brain metastases [47], [48]. In contrast to ANG1005, the liposomal formulation has the additional benefit of a prolonged exposure, i.e. pegylated liposomal doxorubicin has a half-life in humans of 55 h while GRN1005 has a half-life of 3–4 h. Furthermore, a liposomal formulation does not require the active ingredient to be modified, which is the case for conjugates [13].

In conclusion, 2B3-101, as a combination of an existing therapeutic, i.e. pegylated liposomal doxorubicin, with a brain drug delivery platform using glutathione as targeting vector, results in a better drug delivery as well as a safe and efficacious treatment option for brain cancer. These initial promising results have already led to the clinical development of 2B3-101: a phase I/IIa clinical study in patients with solid tumors and brain metastases or recurrent malignant glioma is currently ongoing (clinicaltrials.gov NCT01386580).

## Supporting Information

Figure S1 **Flow cytometry analysis of liposomal doxorubicin-treated hCMEC/D3 cells.** (A) Cells incubated for 5.5 hrs with medium or 2B3-101 or pegylated liposomal doxorubicin (PEG-lipo-DOX), both dosed at 450 µg HSPC per ml, presented similar forward and side scatter (FSC/SSC) plots by FACS, suggesting a lack of cytotoxicity. Histograms of the FL2 channel for the different gates demonstrate the differences of MFI between the different treatments and the increased fluorescence of cells incubated with 2B3-101 compared to pegylated liposomal doxorubicin. (B) Comparative FL2 channels fluorescence histogram of medium- (grey), 2B3-101- (black line) or pegylated liposomal doxorubicin-treated (grey line) hCMEC/D3 cells.
(TIFF)

Table S1 **Number of animals throughout the efficacy studies in a mouse model of glioblastoma.** Animal numbers are given at start of treatment (day 0) and from day 10 onwards. Animals were sacrificed when they lost more than 20% of their body weight, which was the result of the progressive deterioration of their condition.
(DOCX)
