# Supplementary figures and images for: Pharmacokinetics, Brain Delivery, and Efficacy in Brain Tumor-Bearing Mice of Glutathione Pegylated Liposomal Doxorubicin (2B3-101)
Source: PLoS One. 2014 Jan 8;9(1):e82331. doi: 10.1371/journal.pone.0082331 (PMC3885379; doi:10.1371/journal.pone.0082331)

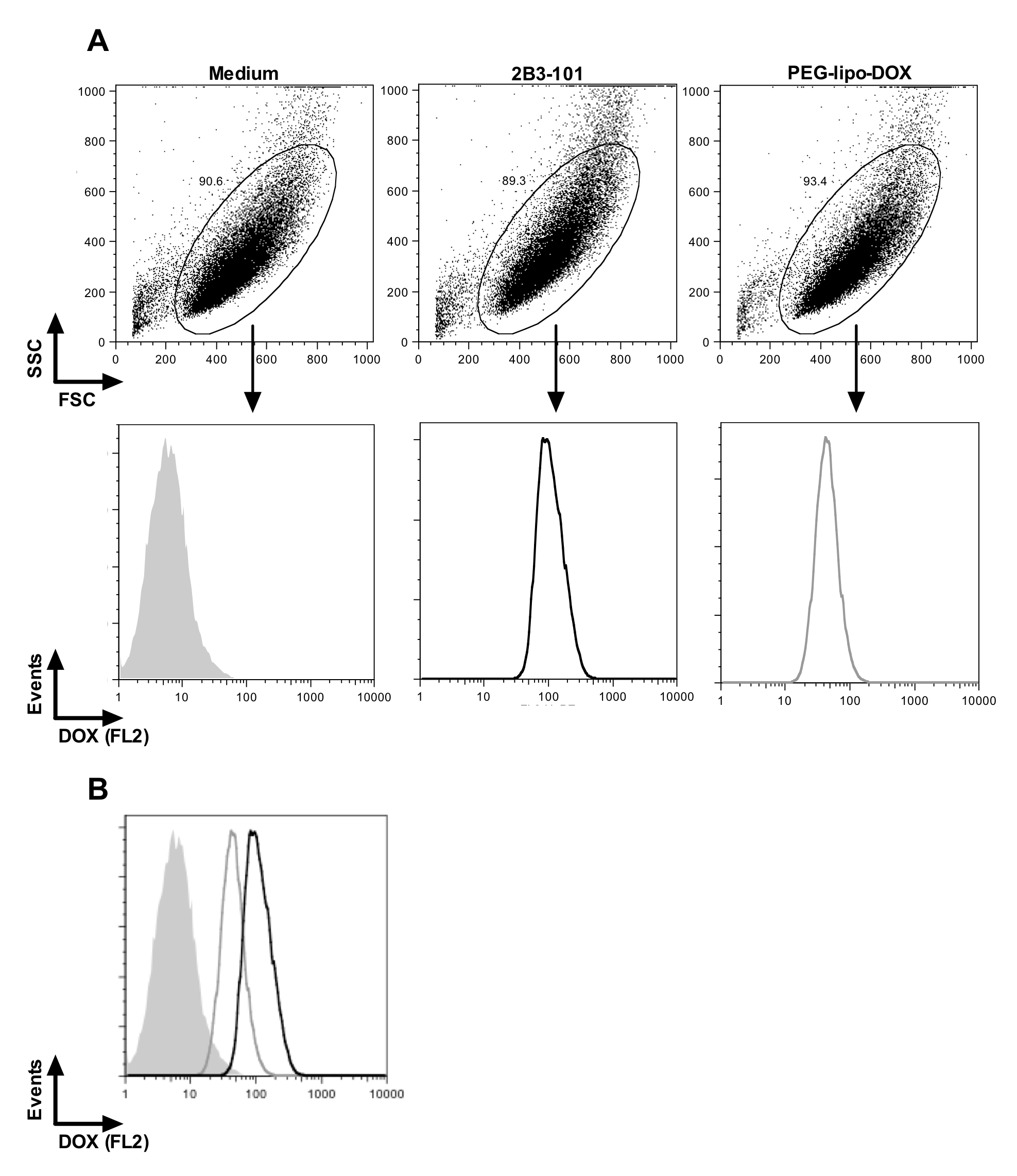

Supplement: Figure S1 — Flow cytometry analysis of liposomal doxorubicin-treated hCMEC/D3 cells. (A) Cells incubated for 5.5 hrs with medium or 2B3-101 or pegylated liposomal doxorubicin (PEG-lipo-DOX), both dosed at 450 µg HSPC per ml, presented similar forward and side scatter (FSC/SSC) plots by FACS, suggesting a lack of cytotoxicity. Histograms of the FL2 channel for the different gates demonstrate the differences of MFI between the different treatments and the increased fluorescence of cells incubated with 2B3-101 compared to pegylated liposomal doxorubicin. (B) Comparative FL2 channels fluorescence histogram of medium- (grey), 2B3-101- (black line) or pegylated liposomal doxorubicin-treated (grey line) hCMEC/D3 cells. (TIFF) [file pone.0082331.s001.tiff]
