# Supplementary material for: Pharmacokinetics, Brain Delivery, and Efficacy in Brain Tumor-Bearing Mice of Glutathione Pegylated Liposomal Doxorubicin (2B3-101)
Source: PLoS One. 2014 Jan 8;9(1):e82331. doi: 10.1371/journal.pone.0082331 (PMC3885379; doi:10.1371/journal.pone.0082331)
Supplement: Table S1 — Number of animals throughout the efficacy studies in a mouse model of glioblastoma. Animal numbers are given at start of treatment (day 0) and from day 10 onwards. Animals were sacrificed when they lost more than 20% of their body weight, which was the result of the progressive deterioration of their condition. (DOCX) [file pone.0082331.s002.docx]

**Supplementary Table 1: Number of animals throughout the efficacy studies in a mouse model of glioblastoma.** Animal numbers are given at start of treatment (day 0) and from day 10 onwards. Animals were sacrificed when they lost more than 20% of their body weight, which was the result of the progressive deterioration of their condition.

|  |  | Start treatment | Day 10 | Day 14 | Day 17 |
| --- | --- | --- | --- | --- | --- |
| *First experiment: treatment once weekly** | | | | | |
| Saline | *n* | 9 | 9 | 9 | 6 |
| Free DOX | *n* | 9 | 9 | 9 | 7 |
| PEG-lipo-DOX | *n* | 9 | 9 | 9 | 9 |
| 2B3-101 | *n* | 9 | 9 | 9 | 9 |
| *Second experiment: treatment twice weekly*** | | | | | |
| Saline | *n* | 14 | 14 | 6 | 0 |
| PEG-lipo-DOX | *n* | 10 | 10 | 8 | 3 |
| 2B3-101 | *n* | 10 | 10 | 8 | 8 |

* Results are depicted in Figure 5A (Bodyweight) and Figure 6 (Tumor growth). Animals received once-weekly IV administrations of saline, 2B3-101, pegylated liposomal doxorubicin (PEG-lipo-DOX) or free doxorubicin (free DOX), all at a 5 mg/kg doxorubicin equivalent. The final administration was on Day 14.

** Results are depicted in Figure 5B (Bodyweight) and Figure 7 (Tumor growth and survival). Animals received twice-weekly IV administrations of saline, 2B3-101, or pegylated liposomal doxorubicin (PEG-lipo-DOX), all at a 5 mg/kg doxorubicin equivalent. The final administration was on Day 10.
